# Supplementary material for: Validation of MSIntuit as an AI-based pre-screening tool for MSI detection from colorectal cancer histology slides
Source: Nat Commun. 2023 Nov 6;14:6695. doi: 10.1038/s41467-023-42453-6 (PMC10628260; doi:10.1038/s41467-023-42453-6)
Supplement: Supplementary file 1 — Supplementary Information [file 41467_2023_42453_MOESM1_ESM.pdf]

Supplementary Information

Supplementary methods

Slide selection. Guidelines regarding slide selection defined to guide pathologists for the use of MSIntuit in clinical practice were to follow the maximum number of the following criteria: the slide with the largest surface of tumour tissue, the slide with the most invasive tumour, the slide with the least necrosis, the slide must not contain preparation artefacts (staining artefacts, folds on the fabric cut, residual air or water bubbles, traces of marker, damaged coverslips, scanning artefacts).

Bland-Altman plot to assess inter-scanner reliability. The Bland-Altman plot was also used (Supplementary figure 3) to assess the agreement between DP200 and UFS prediction scores, and the 95% limits of agreement (LoA) were calculated as mean±1.96 standard deviation (SD) of the difference (DP200 Score - UFS score) (Supplementary table 12). A p-value < 0.05 was considered statistically significant.

ICC and Cohen's Kappa to assess inter-scanner reliability. The intraclass Correlation Coefficients (ICC) was also used to measure the agreement of the continuous predictions of the same slides digitised with UFS and DP200 scanners. Specifically, we used a single-measurement (i.e. same patient), absolute agreement, two-way mixed effects (fixed raters i.e. scanners across all targets i.e. patients) model which corresponds to the ICC(A, 2) form.[1] The ICC value indicates how much of the score variance can be explained by random effects (subjects) and not fixed effects (scanners). An ICC below 0.5 indicates poor reliability, an ICC between 0.5 and 0.75 indicates moderate reliability, an ICC between 0.75 and 0.9 indicates good reliability, and an ICC above 0.9 indicates excellent reliability.[2] A Cohen's kappa under 0.2 indicates slight agreement, 0.21 to 0.40 indicates fair agreement, 0.41–0.60 indicates moderate agreement, 0.61–0.80 indicates substantial agreement, and 0.81 to 1.0 indicates almost perfect agreement.[3]

Slide registration. WSIs of the samples obtained with the DP200 and UFS scanners were not perfectly aligned because of each scanner's principles of operations (orientation of the objective, automatic cropping of empty regions, etc). To compare tile individual scores across the two scanners (figure 2E), we therefore used an image registration process to make sure the local regions of one slide match the local regions of its counterpart digitised with the other scanner. This registration process was done using the Elastix and Transformix softwares.[4,5] Non-rigid registration parameters were first computed on sub-sampled WSIs (8µm per pixel), optimising the Mattes Advanced Mutual Information on ten consecutive levels of resolution. Those parameters were finally applied to the high resolution UFS WSI in order to obtain aligned WSIs at identical resolutions.

Interpretability analysis. For each tile, four pathologists were asked to annotate the presence of the following histology criteria: normal, fibrosis, inflammation, muscle/vessels, tumour, necrosis, mucin. Majority voting was used to settle disagreements between pathologists and annotations of a 5th pathologist (D.E.) were used for cases where two pathologists disagreed with the two others.

Software and libraries used. The experiments were carried out with python (version 3.8) and made use of the following packages: torch (version 1.11), torchvision (0.12.0), numpy (version

1.19.5), scikit-learn (version 0.24.1), pandas (version 1.4.3), openslide-python (1.1.2), matplotlib (version 3.5.1), scipy (version 1.7.3).

Supplementary figures

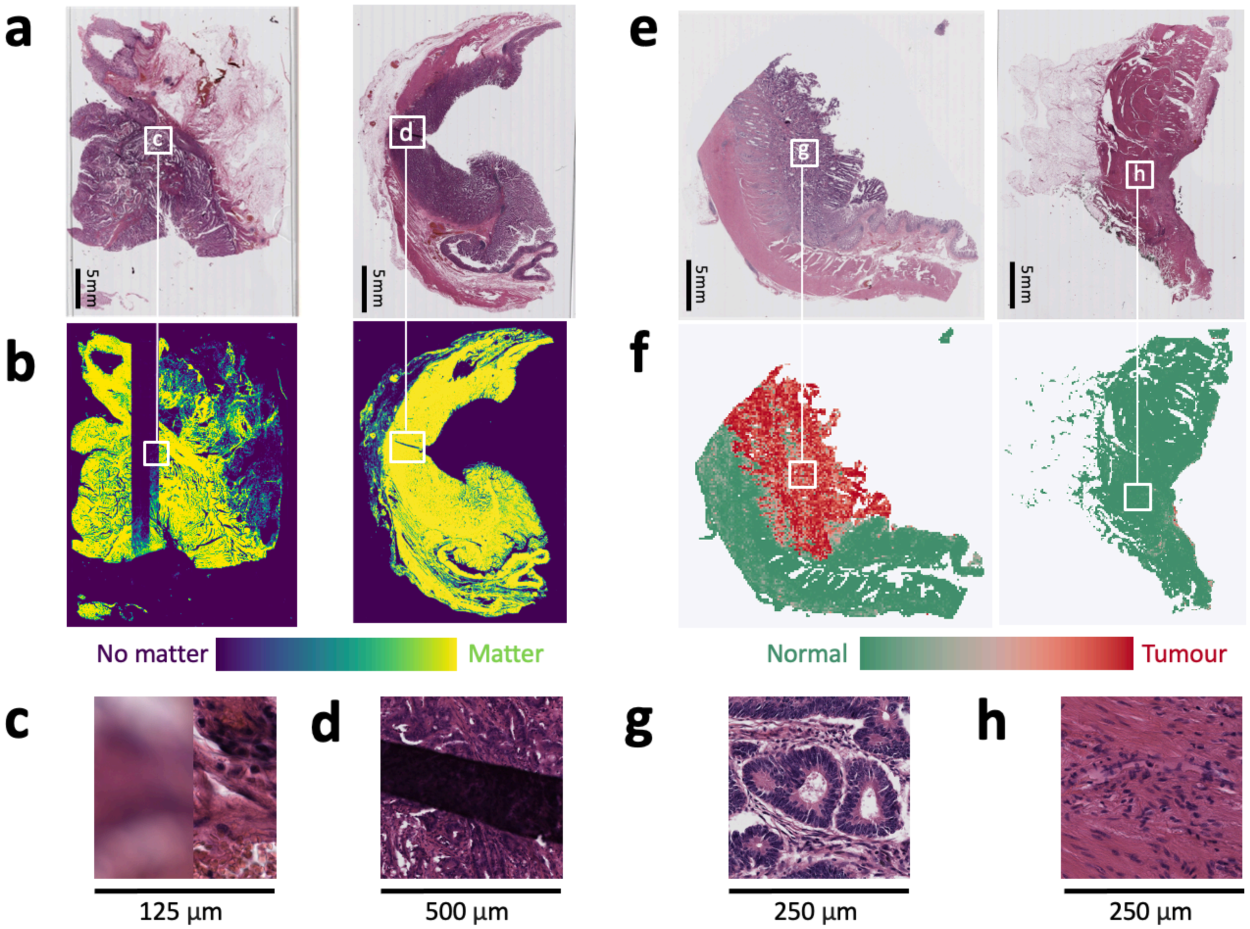

Supplementary figure 1. Quality Check.

a) Left: slide with a blurry strip due to a digitisation issue, not noticeable at low resolution, right : slide with a tissue fold. b) Matter detection heatmaps of the UNet neural network integrated in MSIntuit's preprocessing and QC procedures. Blurry regions (left) and tissue fold (right) are not detected as matter. c), d) Zoomed-in images of blurry and tissue fold regions. e) Slide with abundant tumour tissue that passed QC (left), slide with too few tumour tissue (<500 tumour tiles) that did not pass QC. f) Corresponding tumour heatmaps obtained with a tumour classifier part of MSIntuit's QC procedure. g), h), Zoomed-in images of tumour (left) and (normal) regions of left and right slide, respectively.

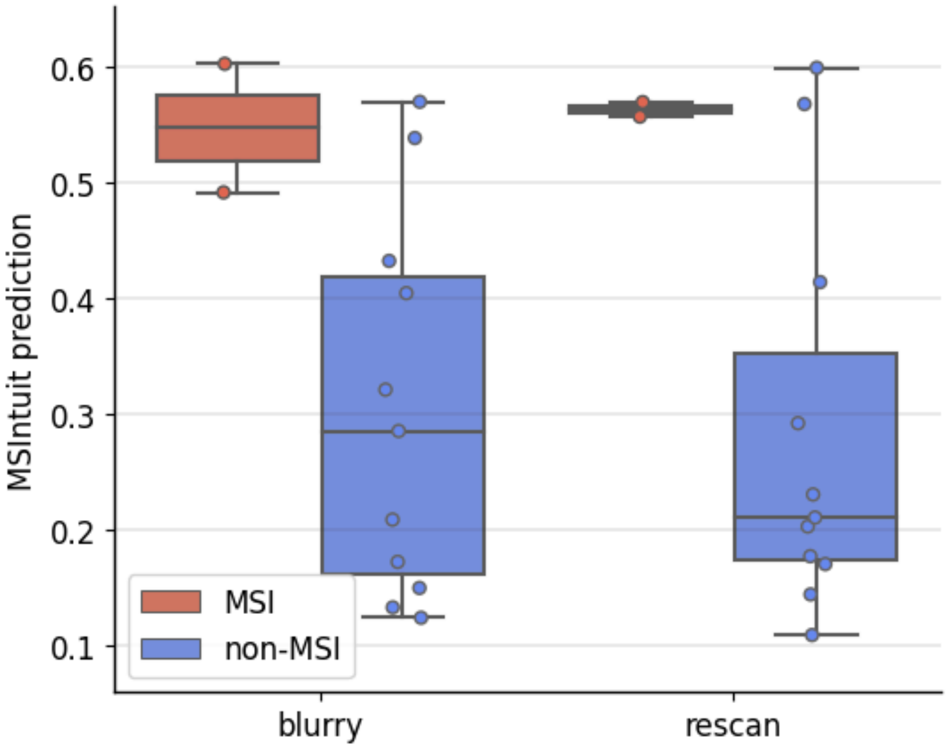

Supplementary figure 2. MSIntuit predictions on slides with large blurry areas and their rescanned counterparts.

We looked at the model predictions of the slides that displayed large blurry areas, which were detected during the QC step (n=13 samples). We compared them against the predictions of slides that were rescanned. Median prediction for blurry (respectively rescanned) slides was of 0.29 (respectively 0.21) for MSS cases and 0.55 (respectively 0.56) for MSI cases. Source data are provided as a Source Data file.

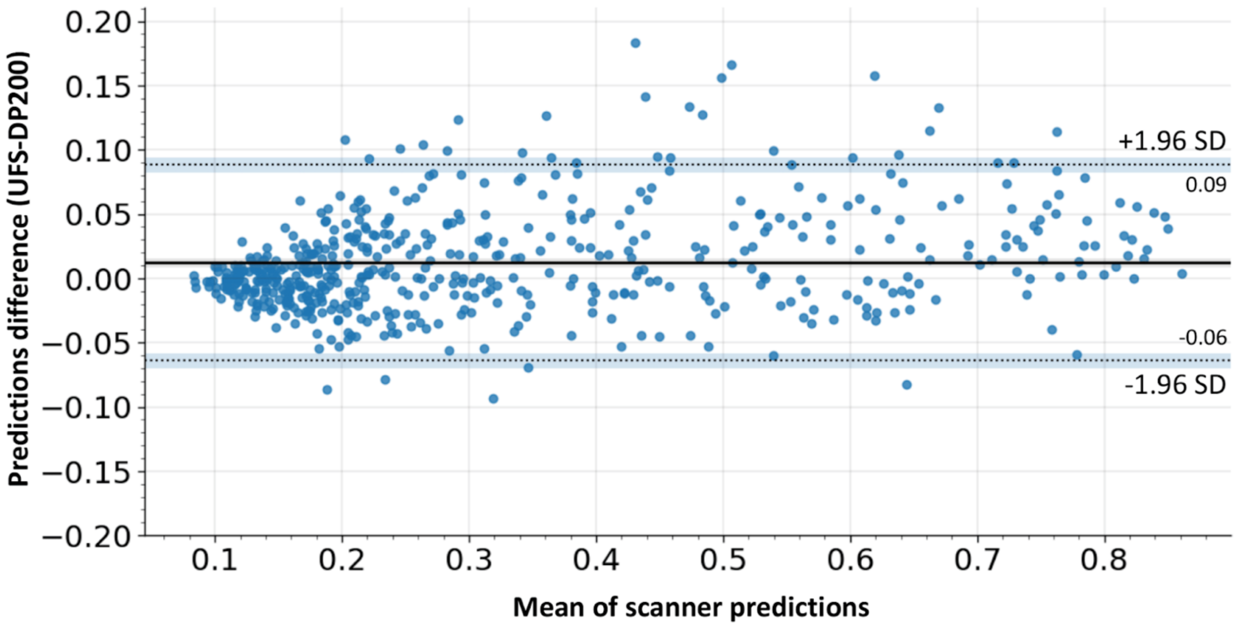

Supplementary figure 3. Bland-Altman plot for inter-scanner reliability.

A Bland-Altman plot to analyse the agreement of MSIntuit predictions on UFS and DP200 scanners by looking at the mean inter-scanner difference of prediction scores (n = 540 samples). A relatively low prediction score variability was observed with an overall mean inter-scanner score difference of 0.01 (where the MSIntuit score can vary between 0 and 1) with a limit of agreement 95% confidence interval ranging from -0.06 to 0.09. Source data are provided as a Source Data file.

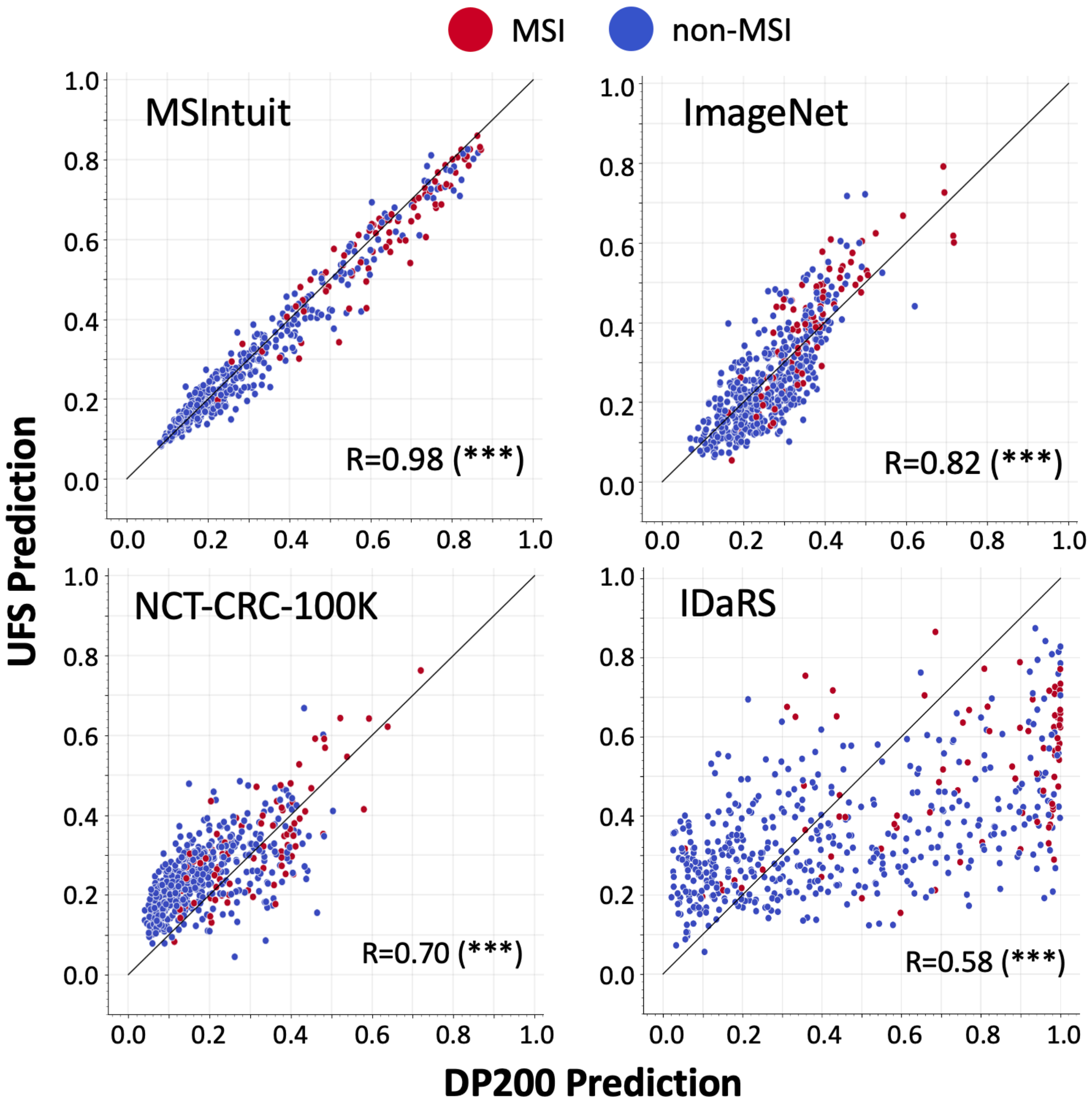

Supplementary figure 4. Robustness to scanner comparison of MSIntuit and other machine learning methods.

Correlation of the predictions on the same n=540 slides digitised on the UFS/DP200 scanners resulted in a Pearson's correlation of 0.98 (two-sided t-test $p<0.001$), 0.82 ($p<0.001$), 0.70 ($p<0.001$) and 0.58 ($p<0.001$) for MSIntuit, ImageNet, NCT-CRC-100K and iDaRS methods, respectively. Source data are provided as a Source Data file.

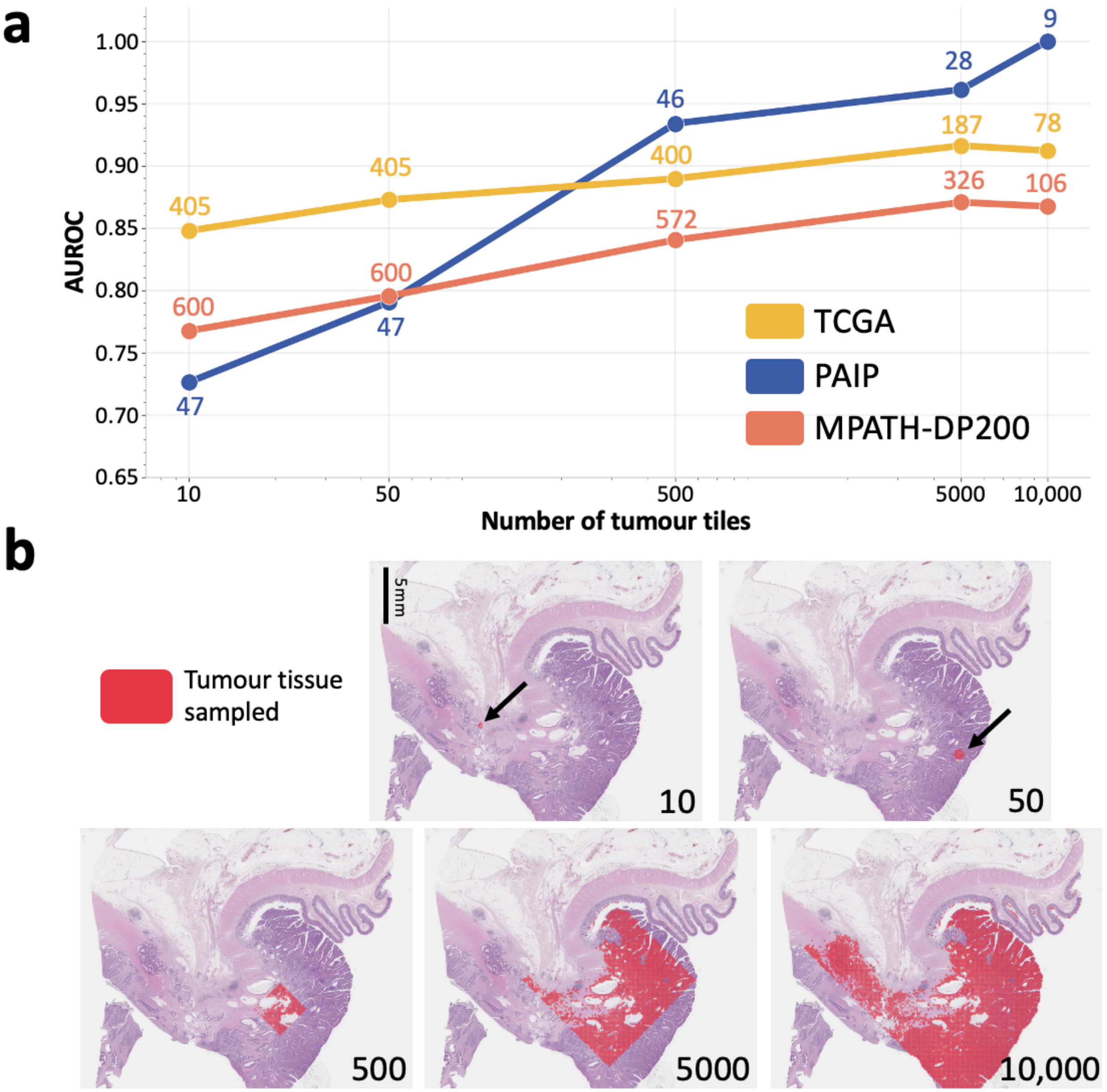

Supplementary figure 5. Impact of amount of tumour on the model.

To assess the minimum amount of tumour on the slide needed to ensure MSIntuit yields good performance, we looked at how the number of tumour tiles impact the results obtained on TCGA and PAIP cohorts before performing the blind-validation. a) For a number *x* being 10, 50, 500, 5000, 10000, we randomly selected an area of x tumour tiles for each slide and performed the prediction on it. Slides with less than x tumour tiles were discarded. Number of slides that contain at least x tumour tiles are displayed next to each point. X-axis is in log scale, b) Example of tumour areas selected, for different numbers of tumour tiles (bottom right corner of each image).

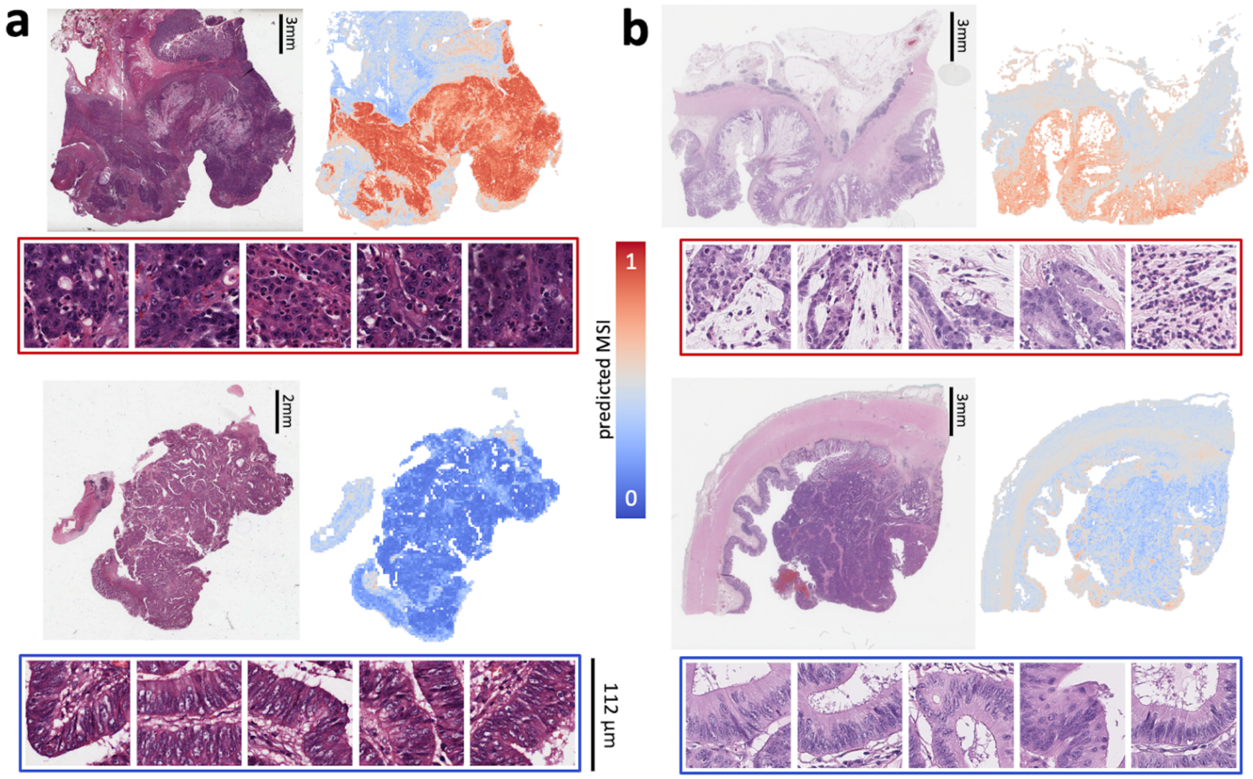

Supplementary figure 6. Model's interpretability on TCGA & PAIP cohorts.

Heatmaps of the tool with corresponding most predictive tiles of a representative MSI case (top) and a pMMR/MSS case (bottom) of a) TCGA cohort, b) PAIP cohort.

Supplementary tables

Supplementary table 1: Performance comparison of MSIntuit against several other pre-training approaches.

We compared the SSL-base pre-training of our feature extractor against two different pre-trainings: ImageNet pre-training and NCT-CRC-100K pre-training. The first one consists of using a feature extractor pre-trained on ImageNet dataset in a supervised fashion. The second one consists of using a feature extractor pre-trained in a supervised fashion on NCT-CRC-100K, a dataset of 100,000 colorectal cancer images, to predict nine tissue classes.[6] Apart from the feature extraction, the same pipeline was used for all methods (QC, downstream model etc ..). In order to provide a fair comparison against MSIntuit, we benchmarked both the last block and penultimate block of the architecture, as the higher layer neurons of such networks are known to be too specialised for their original task.[7] AUROCs obtained on TCGA (cross-validation), PAIP, MPATH-DP200 and MPATH-UFS cohorts are reported in the table below.

Pre-training dataset	Method	Block	TCGA	PAIP	MPATH-DP200	MPATH-UFS
ImageNet	Supervised	Penultimate	0.80 +- 0.05	0.92 [0.84-0.97]	0.79 [0.74-0.83]	0.78 [0.73-0.83]
		Last	0.81 +- 0.04	0.88 [0.73-0.98]	0.78 [0.73-0.82]	0.73 [0.67-0.77]
NCT-CRC-100K	Supervised	Penultimate	0.79 +- 0.06	0.81 [0.67-0.92]	0.79 [0.75-0.83]	0.68 [0.62-0.73]
		Last	0.77 +- 0.04	0.72 [0.56-0.86]	0.71 [0.66-0.76]	0.61 [0.56-0.67]
TCGA	Self-supervised (MSIntuit)	Last	0.93 +- 0.03	0.96 [0.90-0.99]	0.88 [0.84-0.91]	0.87 [0.83-0.90]

Supplementary table 2: Performance comparison of MSIntuit against iDaRS.

We compared the performance of MSIntuit against a ResNet34 from TIAToolbox library, trained on colorectal cancer slides from TCGA using iDaRS methodology. [8,9] Performances of these models are reported in the table below on three external datasets (PAIP, MPATH-DP200 and MPATH-UFS).

	PAIP	MPATH-DP200	MPATH-UFS
iDARS (TIAToolbox)	0.86 [0.75-0.94]	0.80 [0.76-0.85]	0.76 [0.71-0.81]
MSIntuit	0.96 [0.90-0.99]	0.88 [0.84-0.91]	0.87 [0.83-0.90]

Supplementary table 3: Training the model on FFPE slides only versus FFPE and frozen slides of TCGA-COAD.

Both FFPE and snap-frozen slides are available for most patients of the TCGA-COAD dataset, the dataset we used for training. Although MSIntuit is intended to be used on FFPE slides, we found that using frozen slides in addition to FFPE ones during Chowder training slightly improved performance when validating the tool on FFPE samples, likely because the Chowder model gained robustness with this augmentation strategy all the while doubling our sample size. In the table below, we compared the performance of two models : one model trained using only FFPE slides, and another model which uses both FFPE and frozen slides for training (MSIntuit). In the table below, we display the results obtained when validating on FFPE slides of TCGA-COAD (cross-validation), PAIP and MPATH-DP200 datasets (external validation).

Cohort	Metric	FFPE Only	FFPE & Frozen (MSIntuit)

TCGA-COAD	AUROC	0.91 +- 0.02	0.93 +- 0.03
PAIP	AUROC	0.97 [0.90-0.99]	0.97 [0.90-0.99]
MPATH-DP200	AUROC	0.88 [0.84-0.90]	0.88 [0.84-0.91]
	Sensitivity	0.94 [0.90-0.98]	0.98 [0.95-1.00]
	Specificity	0.57 [0.53-0.60]	0.46 [0.42-0.50]
MPATH-UFS	AUROC	0.86 [0.82-0.89]	0.87 [0.83-0.90]
	Sensitivity	0.96 [0.92-0.99]	0.96 [0.91-0.99]
	Specificity	0.42 [0.38-0.46]	0.47 [0.43-0.51]

Supplementary table 4: Training/Testing on tumour regions only.

Even though known MSI-related features are found only within tumour regions, we found that applying our model on the whole slide yielded slightly better results. In the table below, we compare the performance of two models : one model trained and validated using only tumour regions of the slide, and MSIntuit which keeps the whole slide for training and validation. tumour regions were defined using a tumour detection model (see section Quality Checks of Material and Methods).

Cohort	Metric	Tumour Only	Whole slide (MSIntuit)
TCGA-COAD	AUROC	0.90 +- 0.03	0.93 +- 0.03
PAIP	AUROC	0.94 [0.85-0.99]	0.97 [0.90-0.99]
MPATH-DP200	AUROC	0.88 [0.85-0.91]	0.88 [0.84-0.91]
	Sensitivity	0.96 [0.91-0.99]	0.98 [0.95-1.00]
	Specificity	0.45 [0.41-0.48]	0.46 [0.42-0.50]

Supplementary table 5: Performance to detect unusual isolated losses of PMS2 and MSH6.

We assessed the ability of MSIntuit to detect unusual isolated losses of PMS2 and MSH6 on MPATH-DP200 and MPATH-UFS cohorts. Sensitivity for each protein loss is given in the table below.

Loss	# MPATH-DP200 cases with isolated loss	# MPATH-UFS cases with isolated loss	Sensitivity on MPATH-DP200	Sensitivity on MPATH-UFS
PMS2	10	10	0.91 [0.7-1.0]	0.91 [0.85-0.95]
MSH6	3	5	0.67 [0.0-1.0]	0.72 [0.54-0.86]

<u>Supplementary table 6</u>: Ablation study of QC step on MPATH-DP200.

We conducted an ablation study on the MPATH-DP200 cohort of the two QC steps (tumour check and blurry check). Ablation of tumour check: we kept slides with too few tumour instead of discarding them. This means that 28 slides with small tumour areas were added to the validation cohort. Ablation of blurry check: we kept the slides with large blurry areas (n=13), instead of using the rescanned version. Model performance with these experiments can be found below.

	n	AUC	Sensitivity	Specificity	NPV
QC (tumour and blurry check, baseline)	537	0.88 [0.84-0.91]	0.98 [0.95-1.0]	0.46 [0.42-0.50]	0.99 [0.98-1.0]
No tumour check	565	0.86 [0.82-0.89]	0.96 [0.91-0.99]	0.45 [0.42-0.49]	0.98 [0.97-1.0]
No blurry check	537	0.88 [0.85-0.91]	0.98 [0.95-1.0]	0.46 [0.42-0.50]	0.99 [0.98-1.0]

<u>Supplementary table 7</u>: Univariate analysis of MSPath features on MPATH-DP200.

Distribution of MSPath features for a subset of 202 cases of MSPath DP200 cohort (MSI: n=39, 19%), stratifying by MSI status. Sensitivity and specificity are given for each feature, as well as the distribution of MSIntuit prediction for each subgroup.

Feature	Subgroup	MSI (row %)	non-MSI (row %)	Sensitivity (95% CI)	Specificity (95% CI)	Median MSIntuit prediction (95% CI)
Age at diagnosis	< 50	0	11 (100)	0 (0-0)	93 (90-96)	0.33 (0.15-0.61)
	>= 50	39 (20)	152 (80)			0.25 (0.11-0.79)
Anatomical site	Right-sided	35 (29)	85 (71)	90 (81-97)	48 (42-54)	0.32 (0.11-0.82)
	Left-sided	4 (5)	78 (95)			0.21 (0.12-0.53)

Histological Type	Mucinous or other	3 (30)	7 (70)	8 (2-16)	96 (93-98)	0.58 (0.34-0.84)
	Adenocarcinoma	36 (19)	156 (81)			0.24 (0.11-0.76)
Grade	Poorly differentiated	10 (71)	4 (29)	26 (14-38)	98 (95-99)	0.77 (0.51-0.85)
	Other	29 (15)	159 (85)			0.24 (0.11-0.72)
Crohn-like reaction	Yes	13 (24)	42 (76)	33 (21-46)	74 (69-80)	0.24 (0.12-0.80)
	No	26 (18)	121 (82)			0.26 (0.11-0.78)
Tumour infiltrating lymphocytes	Yes	15 (33)	30 (67)	38 (26-50)	82 (76-87)	0.29 (0.14-0.81)
	No	24 (15)	133 (85)			0.24 (0.11-0.76)

Supplementary table 8: Logistic regression model combining MSPath and MSIntuit classification scores.

We trained a logistic regression to predict the MSI status taking as input the MSPath and MSIntuit binary classification outputs on a subset of cases (n=202) from MPATH-DP200. For each variable, we give the coefficients, standard error, z-value, p-value and 95% confidence interval bounds.

Variable	coef	Std err	z	p	0.025	0.975
Intercept	-6.9986	1.425	-4.910	0.000	-9.792 -	-4.205
MSPath	3.2081	1.035	3.100	0.002	1.180	5.236
MSIntuit	3.4138	1.032	3.307	0.001	1.390	5.437

Supplementary table 9 : Confusion matrix of MSIntuit classification vs MSPath classification.

Below, one can find the assignments of MSPath and MSIntuit on a subset of 202 cases from MPATH-DP200 cohort, stratifying by MSI status (ground truth). Interestingly, 18% (respectively 22%) of the population were misclassified by MSPath (respectively MSIntuit) but correctly classified by MSIntuit (respectively MSPath). A simple dichotomic classifier F(MSPath classification, MSIntuit classification) = 0 if (MSPath or MSIntuit classification is 0) else 1 yielded a Sensitivity of 0.95 and a Specificity of 0.67.

		MSI Status	
MSPath	MSIntuit	non-MSI	MSI
0	MSS-AI	31	0
	Undetermined	35	1
1	MSS-AI	43	1
	Undetermined	54	37

Supplementary table 10: Cohorts description.

	TCGA	PAIP	Medipath (MPATH-DP200 / MPATH-UFS)
Number of patients	434	47	600
Region	United States	South Korea	France
H&E FFPE slides, n	427	47	600
H&E Frozen slides, n	432	-	-
MSI patients, n (%)	78 (18)	12 (26)	123 (21)
dMMR/MSI diagnosis	MSI-PCR	MSI-PCR	MMR-IHC 4-plex, followed by MSI-PCR for indeterminate cases
Scanner	Aperio	Aperio AT2	Ventana DP200 & Phillips Ultra Fast Intellisite
Age at diagnosis, IQR	68 (58-77)	-	74 (64-82)
Well differentiated, n (%)	-	-	219 (39)
Moderately differentiated, n (%)	-	-	296 (53)
Poorly differentiated, n (%)	-	-	46 (8)
Stage 0, n (%)	1 (1)	-	11 (2)
Stage I, n (%)	67 (18)	-	114 (20)
Stage II, n (%)	146 (38)	-	217 (37)
Stage III, n (%)	113 (29)	-	219 (38)
Stage IV, n (%)	56 (14)	-	18 (3)

Supplementary table 11: Performance of MSIntuit repeating threshold decision procedure.

Since the calibration step involves selecting some slides to define an appropriate operating threshold, we analysed how the selection of these slides may impact the model performance. To this end, we repeated the calibration step 1000 times (selecting each time a different set of slides to calibrate the tool, and assessing the performance of the model on the remaining patients). Metrics obtained with this experiment are reported in the table below.

	MPATH-DP200	MPATH-UFS
AUROC	0.88 [0.87-0.89]	0.87 [0.85-0.88]
Sensitivity	0.95 [0.82-1.0]	0.95 [0.84-1.0]
Specificity	0.52 [0.16-0.82]	0.47 [0.14-0.72]

Supplementary table 12**:** Intraclass Correlation Coefficient (ICC).

F: value of the F-test, *df*: degrees of freedom, p-value: two-sided F-test p-value. We analysed inter-scanner reliability by computing the ICC scores. An F-test is performed in order to confirm or not the presence of bias during ICC computation. It is computed as the ratio of the mean square error between measurements over the total mean squared error. The degrees of freedom are an indication of the total number of subjects used in the analysis. As suggested by Liljequist et al., an F-value considerably smaller than the total sample size indicates that biases are weak. [1]

MSI Status	ICC	CI 95% ICC	F	df1	df2	p-value
MSI	0.98	[0.97, 0.99]	51.287	85	85	2.37e-44
Non-MSI	0.99	[0.99, 0.99]	91.096	453	453	3.86e-41
Both	0.99	[0.99, 0.99]	110.852	539	539	1.46e-90

Supplementary references

1. Liljequist, D., Elfving, B. & Skavberg Roaldsen, K. Intraclass correlation - A discussion and demonstration of basic features. *PLoS One* **14**, e0219854 (2019).

2. Koo, T. K. & Li, M. Y. A Guideline of Selecting and Reporting Intraclass Correlation Coefficients for Reliability Research. *J. Chiropr. Med.* **15**, 155–163 (2016).

3. McHugh, M. L. Interrater reliability: the kappa statistic. *Biochem. Med.* **22**, 276–282 (2012).

4. Shamonin, D. P. *et al.* Fast parallel image registration on CPU and GPU for diagnostic classification of Alzheimer's disease. *Front. Neuroinform.* **7**, 50 (2013).

5. Klein, S., Staring, M., Murphy, K., Viergever, M. A. & Pluim, J. P. W. elastix: a toolbox for intensity-based medical image registration. *IEEE Trans. Med. Imaging* **29**, 196–205 (2010).

6. Kather, J. N., Halama, N. & Marx, A. *100,000 histological images of human colorectal cancer and healthy tissue*. (2018). doi:10.5281/zenodo.1214456.

7. Yosinski, J., Clune, J., Bengio, Y. & Lipson, H. How transferable are features in deep neural networks? *arXiv [cs.LG]* (2014).

8. Pocock, J. *et al.* TIAToolbox as an end-to-end library for advanced tissue image analytics. *Commun. Med.* **2**, 120 (2022).

9. Bilal, M. *et al.* Development and validation of a weakly supervised deep learning framework to predict the status of molecular pathways and key mutations in colorectal cancer from routine histology images: a retrospective study. *Lancet Digit Health* **3**, e763–e772 (2021).
