# Supplementary material for: Validation of MSIntuit as an AI-based pre-screening tool for MSI detection from colorectal cancer histology slides
Source: Nat Commun. 2023 Nov 6;14:6695. doi: 10.1038/s41467-023-42453-6 (PMC10628260; doi:10.1038/s41467-023-42453-6)
Supplement: Supplementary file 3 — Reporting Summary [file 41467_2023_42453_MOESM3_ESM.pdf]

nature portfolio

Corresponding author(s): Charlie Saillard

Last updated by author(s): Sep 26, 2023

Reporting Summary

Nature Portfolio wishes to improve the reproducibility of the work that we publish. This form provides structure for consistency and transparency in reporting. For further information on Nature Portfolio policies, see our Editorial Policies and the Editorial Policy Checklist.

Statistics

For all statistical analyses, confirm that the following items are present in the figure legend, table legend, main text, or Methods section.

n/a	Confirmed	
☐	☒	The exact sample size (n) for each experimental group/condition, given as a discrete number and unit of measurement
☐	☒	A statement on whether measurements were taken from distinct samples or whether the same sample was measured repeatedly
☐	☒	The statistical test(s) used AND whether they are one- or two-sided<br>*Only common tests should be described solely by name; describe more complex techniques in the Methods section.*
☒	☐	A description of all covariates tested
☒	☐	A description of any assumptions or corrections, such as tests of normality and adjustment for multiple comparisons
☐	☒	A full description of the statistical parameters including central tendency (e.g. means) or other basic estimates (e.g. regression coefficient) AND variation (e.g. standard deviation) or associated estimates of uncertainty (e.g. confidence intervals)
☐	☒	For null hypothesis testing, the test statistic (e.g. F, t, r) with confidence intervals, effect sizes, degrees of freedom and P value noted<br>*Give P values as exact values whenever suitable.*
☒	☐	For Bayesian analysis, information on the choice of priors and Markov chain Monte Carlo settings
☒	☐	For hierarchical and complex designs, identification of the appropriate level for tests and full reporting of outcomes
☒	☐	Estimates of effect sizes (e.g. Cohen's d, Pearson's r), indicating how they were calculated

Our web collection on statistics for biologists contains articles on many of the points above.

Software and code

Policy information about availability of computer code

Data collection	Slides of TCGA were digitised with an Aperio scanner, at a resolution of 0.25 or 0.5 microns per pixel. Slides of PAIP were digitised with an Aperio AT2 scanner at a resolution of 0.25 microns per pixel. Slides of the validation set were digitised with Ventana DP200 and Philips UFS scanners, at a resolution of 0.25 microns per pixel.
Data analysis	The experiments were carried out with python (version 3.8) and mase use of the following packages: torch (version 1.11), torchvision (0.12.0), numpy (version 1.19.5), scikit-learn (version 0.24.1), pandas (version 1.4.3), openslide-python (1.1.2), matplotlib (version 3.5.1), scipy (version 1.7.3). An implementation of the U-Net is available at https://github.com/milesial/Pytorch-UNet. An implementation of MoCov2 is available at https://github.com/facebookresearch/moco. Finally, an implementation of Chowder algorithm is made available at https://github.com/CharlieCheckpt/msintuit

For manuscripts utilizing custom algorithms or software that are central to the research but not yet described in published literature, software must be made available to editors and reviewers. We strongly encourage code deposition in a community repository (e.g. GitHub). See the Nature Portfolio guidelines for submitting code & software for further information.

Data

Policy information about availability of data

All manuscripts must include a data availability statement. This statement should provide the following information, where applicable:

- Accession codes, unique identifiers, or web links for publicly available datasets
- A description of any restrictions on data availability
- For clinical datasets or third party data, please ensure that the statement adheres to our policy

All images and the associated MSI status for the TCGA cohort used in this study are publicly available at https://portal.gdc.cancer.gov/ and cBioPortal (https://www.cbioportal.org/). Deidentified pathology images and annotations from the PAIP cohort can be obtained via appropriate data access requests at http://www.wisepaip.org/paip. Datasets MPATH-DP200 and MPATH-UFS are the property of Owkin and are available upon request for academic use only.

Human research participants

Policy information about studies involving human research participants and Sex and Gender in Research.

Reporting on sex and gender	*Use the terms sex (biological attribute) and gender (shaped by social and cultural circumstances) carefully in order to avoid confusing both terms. Indicate if findings apply to only one sex or gender; describe whether sex and gender were considered in study design whether sex and/or gender was determined based on self-reporting or assigned and methods used. Provide in the source data disaggregated sex and gender data where this information has been collected, and consent has been obtained for sharing of individual-level data; provide overall numbers in this Reporting Summary. Please state if this information has not been collected. Report sex- and gender-based analyses where performed, justify reasons for lack of sex- and gender-based analysis.*
Population characteristics	*Describe the covariate-relevant population characteristics of the human research participants (e.g. age, genotypic information, past and current diagnosis and treatment categories). If you filled out the behavioural & social sciences study design questions and have nothing to add here, write "See above."*
Recruitment	*Describe how participants were recruited. Outline any potential self-selection bias or other biases that may be present and how these are likely to impact results.*
Ethics oversight	*Identify the organization(s) that approved the study protocol.*

Note that full information on the approval of the study protocol must also be provided in the manuscript.

Field-specific reporting

Please select the one below that is the best fit for your research. If you are not sure, read the appropriate sections before making your selection.

☒ Life sciences ☐ Behavioural & social sciences ☐ Ecological, evolutionary & environmental sciences

For a reference copy of the document with all sections, see nature.com/documents/nr-reporting-summary-flat.pdf

Life sciences study design

All studies must disclose on these points even when the disclosure is negative.

Sample size	For all cohorts, sample sizes were determined based on the maximum number of samples available which respect the inclusion criteria detailed below.
Data exclusions	Inclusion criteria for all cohorts were as follows: unequivocal histological diagnosis of colorectal cancer, available histological slides of resected specimens from the primary tumour, available MSI status.
Replication	The results presented here have been generated using MSIntuit, a reproducible software that obtained CE-marking.
Randomization	Patients of the development cohort were randomly divided for cross-validation into training and validation sets, stratified with respect to their MSI status. No randomization was applied for the indenpendent validation sets.
Blinding	Prediction procedure was performed in a one-shot fashion and blinded to each patient MSI status to avoid the risk of overfitting.<br>Regarding model interpretability : pathologists were independently assigned regions of interest to review and were not able to communicate on their results to each other so that there is no bias in each pathologist review.

Reporting for specific materials, systems and methods

We require information from authors about some types of materials, experimental systems and methods used in many studies. Here, indicate whether each material, system or method listed is relevant to your study. If you are not sure if a list item applies to your research, read the appropriate section before selecting a response.

Materials & experimental systems

n/a	Involved in the study
☐	☒ Antibodies
☒	☐ Eukaryotic cell lines
☒	☐ Palaeontology and archaeology
☒	☐ Animals and other organisms
☐	☒ Clinical data
☒	☐ Dual use research of concern

Methods

n/a	Involved in the study
☒	☐ ChIP-seq
☒	☐ Flow cytometry
☒	☐ MRI-based neuroimaging

Antibodies

Antibodies used	MLH1, MSH2, PMS2 and MSH6 antibodies were used for MMR-IHC.
Validation	For each antibody, the proper staining was verified (proper stained cell and proper localisation of the staining (nucleus, cytoplasm etc...)) by a certified pathologist.

Clinical data

Policy information about clinical studies

All manuscripts should comply with the ICMJEguidelines for publication of clinical research and a completedCONSORT checklist must be included with all submissions.

Clinical trial registration	NA
Study protocol	NA
Data collection	The discovery cohort, denoted TCGA here, is a multicentric cohort of 859 whole slide images (WSI) from 434 patients from the TCGA-COAD database diagnosed in 24 US centres. 427 Formalin-Fixed Paraffin-Embedded (FFPE) and 432 snap frozen H&E-stained WSIs from these patients associated with MSI-PCR status were used to develop our model. The PAIP cohort was used as a development set and comprised colorectal tumour samples of n=47 patients, collected from three centres in South Korea. The MSI status of these patients was determined using MSI-PCR assays. The validation cohort used for the blind validation consisted of 600 anonymised FFPE H&E WSIs of 600 consecutive resected CRC diagnosed at Medipath pathology laboratories (France) in 2017 and 2018. For each patient, one H&E slide was chosen following our guidelines . All slides were digitised using two scanners, Philips UFS (Philips, Amsterdam, The Netherlands) and Ventana DP200 (Roche Diagnostics GmbH, Mannheim, Germany), leading to two sets of 600 WSIs referred to as MPATH-UFS and MPATH-DP200. dMMR status was assessed using MMR-IHC for the four MMR proteins, and confirmed by MSI-PCR for n=33 indeterminate cases (doubt in MMR-IHC interpretation or suspicion of Lynch Syndrome).
Outcomes	NA
